# Piecewise latent growth modeling as the methodological standard for evaluation research

**DOI:** 10.3389/fpsyg.2026.1830316

**Published:** 2026-08-24

**Authors:** Changiz Mohiyeddini

**Affiliations:** 1 Michael Garron Hospital, Toronto East Health Network, Toronto, ON, Canada; 2 Department of Family and Community Medicine, Temerty Faculty of Medicine, University of Toronto, Toronto, ON, Canada

**Keywords:** change measurement, curriculum evaluation, evaluation research, longitudinal methodology, ontology of change, piecewise latent growth model, psychotherapy

## Abstract

Evaluation research across applied domains—clinical intervention, psychotherapy, curriculum reform, organizational change—has long relied on the pre–post difference score as its primary evidence of change. This paper argues that this reliance reflects not merely a methodological habit but a systematic ontological error: the implicit treatment of change as a discrete event rather than a continuous, phase-structured process. We develop a theoretical and methodological case for piecewise latent growth modeling (PLGM) as the appropriate evaluative standard across these domains. Drawing on classical psychometric theory, philosophy of measurement, and the longitudinal modeling literature, we establish the conditions under which difference-score logic fails under realistic measurement conditions. We articulate an explicit processual ontology of change and demonstrate its alignment with theoretical commitments already embedded—but rarely operationalized—in leading evaluation frameworks. We present the formal structure of piecewise latent growth models, including two-piece, multi-piece, and growth mixture extensions, illustrated through worked examples from clinical trial evaluation, psychotherapy research, curriculum evaluation in medical education, and organizational change research. We situate PLGM relative to its principal alternatives—polynomial growth models, multilevel models, and exploratory network approaches—and specify the roles of longitudinal measurement-invariance testing and formal model comparison. We address the practical constraints—sample size, measurement frequency, statistical expertise—that limit uptake in resource-constrained settings and propose tiered implementation pathways calibrated to varying analytic capacity. We conclude with a reporting framework for trajectory-based evaluation studies. The argument rests on a single claim: evaluation research cannot produce interpretively valid evidence of change until it adopts methods whose ontological commitments match the phenomena it studies. The field must transition from asking “Did it work?” to “How did it unfold?”—because only the latter produces evidence worthy of its name.

## 1 Introduction

Evaluation research occupies a structurally paradoxical position in applied science. Across its major domains—clinical trial methodology, psychotherapy outcome research, curriculum evaluation in health professions education, organizational change assessment—the theoretical frameworks that guide intervention design have grown increasingly sophisticated. Randomized controlled trials are nested within implementation frameworks. Psychotherapy protocols specify mechanisms of action with growing precision. Curriculum reform initiatives articulate multi-level theories of learning and professional development. Organizational change models distinguish between first-order and second-order change, between adoption, consolidation, and institutionalization.

And yet the evidentiary practice that anchors evaluation across all these domains has remained stubbornly anchored to a measurement logic that predates our current theoretical understanding of change. In the overwhelming majority of applied evaluation studies, evidence of change is operationalized as a difference score: *D* = Post – Pre. The intervention is delivered, two measurements are taken at arbitrary points in time, one is subtracted from the other, and the result—positive, negative, or negligible—is treated as the answer to the question: did change occur?

This paper argues that this practice is not merely statistically suboptimal. It is ontologically misaligned with the phenomena that evaluation research claims to study. Change, in clinical, therapeutic, educational, and organizational contexts, is not an event. It is a process: a temporally extended, phase-structured trajectory with properties—velocity, curvature, inflection, directionality—that cannot be recovered from any pair of point measurements, regardless of how carefully those measurements are taken. The difference score does not measure this process imprecisely. It misrepresents its nature categorically.

The methodological alternative we develop and advocate is piecewise latent growth modeling (PLGM)—a class of longitudinal structural equation models that explicitly represents change as a sequence of qualitatively distinct trajectory segments, each with its own slope, joined at theoretically or empirically defined transition points. PLGM is not simply a more sophisticated statistical technique. It is the operationalization of a different and more defensible ontology of change—one that evaluation research has long required but rarely employed. Two scope limitations should be stated at the outset and are developed below. First, the argument is addressed to evaluation research specifically—inquiry in which an intervention and a theory of how it produces change are specified in advance—rather than to all longitudinal science. Second, PLGM presupposes, rather than supplies, a defensible and longitudinally invariant measurement model; the case made here is conditional on that prerequisite.

We proceed as follows. Section 2 establishes the psychometric and philosophical case against difference scores. Section 3 articulates the processual ontology of change and examines its alignment with existing evaluation frameworks. Section 4 presents the formal structure of piecewise latent growth models, including its treatment of longitudinal measurement invariance and its relationship to alternative models. Section 5 illustrates PLGM across four applied domains. Section 6 addresses practical constraints and tiered implementation pathways. Section 7 offers a reporting framework for trajectory-based evaluation studies.

## 2 The indefensibility of difference-score logic

### 2.1 The reliability problem

The psychometric case against difference scores is well-established, although—as a recent and important counter-argument makes clear—its scope must be stated with care (Trafimow, 2015). The reliability of a difference score D = Y_2_ – Y_1_ is given by:


$$\begin{array}{l} {\text{ρ}}^{\text{D}}=\left({\text{ρ}}_{11}+{\text{ρ}}_{22}-2{\text{ρ}}_{12}\right)/\left(2-\;2{\text{ρ}}_{12}\right) \end{array}$$

where ρ_11_ and ρ_22_ are the reliabilities of the pre- and post-measures, respectively, and ρ_12_ is their correlation. The operative condition that renders this formula problematic is not simply that component reliabilities are high, but that the pre–post correlation is high relative to the component reliabilities—precisely the condition that well-constructed, stable measures of the same construct across proximate time points are designed to produce. As ρ_12_ approaches the average of ρ_11_ and ρ_22_, the reliability of D approaches zero.

Difference scores are not, however, unreliable as a matter of mathematical necessity. Trafimow (2015) has shown that ρ^*D*^ can be entirely acceptable when the component reliabilities ρ_11_ and ρ_22_ are high and the variance of true change is substantial relative to error variance; further, ρ_12_ is itself bounded above by the (geometric) mean of the component reliabilities, so the degenerate case cannot arise when reliabilities are high and the pre–post correlation is genuinely lower. The argument advanced here is therefore narrower than the strong reading of Cronbach and Furby (1970) but is, I argue, more consequential for applied evaluation. The reliability of D degrades precisely under the conditions that rigorous repeated-measures evaluation is designed to create—stable, well-validated instruments measuring the same construct at proximate occasions in populations exhibiting modest true change—because these are exactly the conditions under which the pre–post correlation approaches the component reliabilities (Collins, 2006; Willett, 1988). A second problem compounds the first: the difference-score reliability formula requires both the component reliabilities and the pre–post correlation, yet, the overwhelming majority of published evaluation studies that rely on difference scores report neither, leaving the reliability of their central quantity unexamined.

Cronbach and Furby (1970) concluded from this that difference scores are analytically problematic in most realistic measurement contexts. Lord (1967) demonstrated the related paradox that two mathematically defensible analytic strategies applied to the same pre–post data can yield opposite substantive conclusions. Willett (1988) documented extensively the ways in which difference-score analyses produce misleading estimates of change, particularly in the presence of regression to the mean. These results have been available for over half a century. Their implications for evaluation practice have been largely ignored.

### 2.2 The variance conflation problem

Beyond reliability, the difference score conflates three analytically distinct sources of variance that evaluation research is specifically designed to disentangle: true change, regression to the mean, and measurement error. True change is what the evaluation claims to detect. Regression to the mean is a statistical artifact produced by extreme baseline scores moving toward the population mean on retest, entirely independent of any intervention effect. Measurement error is random fluctuation in scores attributable to the imprecision of the instrument rather than any real-world change in the construct being measured.

The difference score provides no mechanism for disaggregating these sources. A positive *D* could reflect a genuine intervention effect, an artifact of extreme baseline selection, or the chance alignment of measurement errors at two time points. These possibilities are not merely theoretically distinguishable; they have radically different implications for decisions about intervention effectiveness, resource allocation, and policy. An evaluation methodology that cannot distinguish among them is not merely imprecise. It is epistemically blind to the most important question it is asked to answer.

These three sources do not exhaust the problem. The difference score also presupposes that the measurement scale retains identical properties across occasions—that is, longitudinal measurement invariance. When the relationship between the latent construct and its indicators shifts over time [through item-functioning drift, or through response shift in the form of recalibration of internal standards, reconceptualization of the construct, or reprioritization across its dimensions (Sprangers and Schwartz, 1999; Oort, 2005)], the pre–post difference conflates genuine change with change in the measurement model itself, and the scalar D cannot separate the two. This fourth source of variance is arguably the most insidious, because it is undetectable within the very two-wave design that produces the difference score. As Section 4 shows, it is directly testable within a latent-variable growth model and only there.

These statistical failures are not merely technical nuisances; they are the inevitable consequence of trying to represent a multi-faceted process with a single number—the empirical signature of an ontological mistake that the remainder of this paper addresses.

### 2.3 The philosophical dimension: what difference scores claim

Borsboom (2005) identified the deeper philosophical problem: psychological measurement can only be said to measure a construct if variation in the construct causally produces variation in instrument scores. The difference score, as a derived quantity, inherits this requirement for both its components but adds a further one: the arithmetic operation of subtraction must itself be interpretable as a representation of a real-world process. That is, *D* = Post – Pre is interpretable as a measure of change only if change is the kind of thing that arithmetic subtraction can represent—a discrete, one-dimensional displacement from a fixed point. Michell (1999) extends this line of reasoning: the assignment of numbers does not constitute measurement; the structure of numbers must correspond to the structure of the property being quantified. If change is a multi-dimensional, temporally extended, phase-sensitive process, then a scalar subtraction does not measure it. It replaces it with something else and calls that something else by the same name.

These measurement-theoretic concerns apply to any quantitative model of change, piecewise growth models included, and two consequences follow. First, the concerns raised by Borsboom (2005) and Michell (1999)—whether the construct causally produces variation in its indicators, and whether the scale supports the arithmetic operations performed on it—are prerequisites for any analysis of quantitative change, and PLGM inherits rather than dissolves them. A poorly validated instrument analyzed within a piecewise growth model yields invalid phase-specific slopes just as surely as it yields an invalid difference score; PLGM does not repair the measurement-theoretic deficiencies of difference scores. Second, two logically separate claims that the difference-score literature tends to run together must be distinguished. The measurement-theoretic claim concerns whether a defensible quantitative scale exists at all and is preserved across occasions; this is the province of Borsboom and Michell, and it is addressed not by the choice between difference scores and growth models but by validation and by longitudinal measurement-invariance testing (Section 4). The ontological claim—the one this paper advances—concerns what is true given a defensible scale: that change in applied domains is a phase-structured trajectory rather than a scalar displacement, and that an analytic model whose structure represents only displacement discards information that is theoretically and practically consequential. The case for PLGM attaches to this second claim; the measurement-theoretic prerequisite is acknowledged, addressed where addressable through invariance testing, and otherwise held constant across the comparison.

## 3 A processual ontology of change

### 3.1 Event ontology vs. process ontology

The philosophical literature on change distinguishes two fundamental ontological positions (for an overview see Rescher, 1996; on the corresponding distinction between event-based and process-based theories of causation see Salmon, 1984). An event ontology treats change as a discrete transition: a system moves from state S1 to state S_2_, and the change is fully characterized by the difference between those states. On this view, the pre–post comparison is, in principle, adequate: if states are measured accurately, their difference captures what happened. A process ontology, by contrast, treats change as a continuous, temporally extended trajectory: the system does not simply jump from S1 to S_2_ but follows a path through state-space, and the properties of that path—its phases, inflection points, velocity, and directionality—are themselves theoretically meaningful and practically consequential. A parallel distinction has long been emphasized in the developmental and longitudinal-methods literatures, where the analysis of change is understood as inseparable from a theoretically grounded model of its temporal architecture (Collins, 2006; Nesselroade, 1991).

The choice between these ontologies is not merely philosophical; it determines what counts as a measurement and what counts as evidence. Under an event ontology, two time points are sufficient for evidence of change. Under a process ontology, two time points are sufficient only to establish displacement—a much weaker claim that does not speak to the shape, sustainability, or mechanism of the process that produced it.

A piecewise model is, like the difference score, ultimately built from a finite set of measurement occasions, and estimating several segments rather than one does not by itself secure a more realistic ontology. In this superficial sense PLGM might appear simply to substitute “more events” for “two events.” Yet this framing obscures what is in fact being claimed. No observational method dissolves the discreteness of measurement; data are always sampled at occasions. What distinguishes a trajectory model from a displacement model is not the number of occasions sampled but the logical type of the quantity inferred from them. A difference score reports a single scalar contrast between two states; a piecewise growth model estimates rates of change—slopes—and changes in those rates across phases. A rate is not an event: it is a process-level quantity that characterizes how a system is moving through state-space, and it cannot be expressed as a contrast between two static states regardless of how many such contrasts are computed (cf. Salmon, 1984, on process-based theories of causation). The ontological gain of PLGM is therefore not that it accumulates more events but that the parameters it estimates—phase-specific velocities, the locations of transitions, and their heterogeneity across individuals—are categorically different from the displacement a difference score reports. How many pieces a given trajectory warrants is then an empirical and theoretical question to be settled by model comparison (Sections 4 and 5), not the source of the ontological claim.

### 3.2 The processual nature of change in applied domains

Change in the domains that applied evaluation research studies is, without exception in any theoretically sophisticated account, processual rather than event-like. In clinical intervention, treatment effects in chronic disease management, rehabilitation, and preventive medicine unfold across time in non-linear, phase-sensitive trajectories. The Transtheoretical Model articulates five qualitatively distinct phases with different psychological and behavioral properties (Prochaska and DiClemente, 1983). A pre–post comparison that brackets these phases is blind to the phenomenon's most important structural features.

In psychotherapy, the early response literature established that the majority of symptom reduction in effective treatments occurs in the first few sessions, followed by diminishing returns and, in some cases, consolidation or relapse (Baldwin et al., 2009; Howard et al., 1986). A therapy that produces rapid early improvement followed by relapse has a radically different clinical profile—and warrants radically different clinical decisions—from one that produces gradual, sustained improvement, even if the total pre–post displacement is identical.

In curriculum evaluation and health professions education, the formation of clinical competence and professional identity is a developmental trajectory subject to plateaus, apparent regression under novel challenge, and non-linear acceleration when multiple learning streams converge. An evaluation that treats a pre-course and post-course measurement as sufficient evidence of educational change is not measuring the developmental process; it is substituting for it a fiction of convenience.

In organizational change, models such as Kotter's eight-stage change model (Kotter, 1996), Lewin's unfreeze–change–refreeze framework, and complexity-theoretic accounts of organizational transformation all represent change as a sequence of qualitatively distinct phases with different dynamics, resistances, and sustainability conditions. Pre–post comparison collapses these phases into a single displacement, rendering invisible the question of whether the organization has genuinely transformed or merely temporarily adjusted under evaluation pressure.

### 3.3 The mismatch between framework ontology and evaluative practice

A striking feature of applied evaluation research is that theoretical frameworks guiding intervention design are frequently processual in their explicit commitments, while the evaluative methods used to assess those frameworks are uniformly event-based. The Consolidated Framework for Implementation Research (CFIR; Damschroder et al., 2009) conceptualizes implementation as a multilevel, dynamic process unfolding across time—and its phases (exploration, adoption, implementation, and sustainment) are precisely the kind of qualitatively distinct trajectory segments that PLGM is designed to model. Where CFIR articulates adoption as a temporally distinct phase with different facilitative conditions from sustainment, PLGM operationalizes this by estimating phase-specific slopes whose magnitudes and predictors can differ across implementation stages. Proctor et al.'s (2011) outcome taxonomy distinguishes adoption, fidelity, sustainability, and penetration—all trajectory-sensitive properties that cannot be indexed by a single post-minus-pre calculation. Theory-of-Change frameworks—the explicit causal-pathway models used in organizational and educational program evaluation to specify how inputs are expected to produce outcomes through a sequence of intermediate states—articulate causal pathways that are explicitly temporal and sequential; a PLGM knot point is, in structural terms, the operationalization of the causal transition point that such diagrams represent graphically but evaluation designs rarely model statistically.

This mismatch reflects the institutional incentive structure of applied evaluation: the difference score is quick to compute, easy to communicate, and produces clean, legible findings. The costs—systematic misrepresentation of the phenomena being studied, systematic production of misleading evidence, systematic underestimation of intervention complexity—are borne not by those who produce the evaluations but by those who act on their conclusions.

## 4 Piecewise latent growth modeling: structure, logic, and extensions

### 4.1 From latent growth curves to piecewise specification

Latent Growth Curve Modeling (LGCM), developed within the structural equation modeling tradition (McArdle and Epstein, 1987; Meredith and Tisak, 1990; for reviews, see McArdle, 2009; Bollen and Curran, 2006), represents change as a continuous trajectory by estimating latent growth parameters—an intercept factor η_*I*_ representing initial level and a slope factor η_*s*_ representing rate of change. The individual-level model is:


$$\begin{array}{l} {\text{y}}_{\text{it}}={\text{η}}_{\text{I},\text{i}}+{\text{η}}_{\text{S},\text{i}}{\text{λ}}_{\text{t}}+\;{\text{ε}}_{\text{it}} \end{array}$$

In this expression, *y*_*it*_ is the observed score for individual *i* at occasion *t*. The latent factors are person-specific: η_I, i_ is individual *i*'s latent intercept, representing that individual's level at the reference occasion (the time point whose loading is fixed to zero), and η_S, *i*_ is individual *i*'s latent slope, representing that individual's rate of change. The fixed time score λ_*t*_ locates occasion t along the trajectory, and ε_*it*_ is the occasion-specific residual. Across the sample, η_I, *i*_ has an estimated mean μ_*I*_ and variance σ^2^_I_ (the average level and individual differences in level); η_S, *i*_ has an estimated mean μ_*S*_ and variance σ^2^_S_ (the average rate of change and individual differences in rate); and the two factors are permitted to covary.

Standard LGCM assumes a single slope factor adequately characterizes the entire observation period. Whether this single-slope assumption is adequate is an empirical question to be settled by model comparison, not by stipulation. I argue, however, that in the applied evaluation contexts surveyed in Section 3—where the guiding theories of change explicitly posit qualitatively distinct phases (for example, the early-response and consolidation phases of psychotherapy, or the adoption and sustainment phases of implementation)—a single linear slope is frequently a poor representation of the theorized process and should be retained only when a piecewise alternative fails to improve fit. The claim is thus conditional on the substantive theory and testable against the data, rather than a blanket assertion that linear growth is generally indefensible.

Piecewise Latent Growth Modeling (PLGM) relaxes this assumption by specifying two or more distinct growth processes, each operating over a defined phase of the observation window, joined at one or more knot points κ (see Glossary). The loading matrix for a two-piece linear model with knot at time point 3 of five occasions might be coded:

Phase 1 loadings: [0, 1, 2, 2, 2]; Phase 2 loadings: [0, 0, 0, 1, 2]

This coding estimates not one rate of change but two, each with its own mean, variance, and covariance with other model parameters.

### 4.2 Knot point specification: theory-driven vs. data-driven

The theory-driven approach locates the knot at a transition point specified by the theoretical model of the intervention. The data-driven approach estimates the knot location as a free parameter.

These two approaches occupy different epistemic positions. Theory-driven knot specification is the preferred default precisely because it tests a substantive hypothesis: the knot location is a prediction derived from the theory of change, and its fit is informative about that theory. Data-driven knot estimation is appropriate in two bounded circumstances. The first is when the theory of change commits to the existence of a phase transition but is genuinely silent on its timing; here, freeing the knot operationalizes a theoretical commitment—that a transition exists—while declining to over-specify a parameter the theory does not constrain. The second is frankly exploratory analysis, whose results are reported as hypothesis-generating and as requiring confirmation in independent samples. What is not defensible under the processual ontology advocated here is data-driven knot estimation in the absence of any theoretical commitment to a phase structure: that practice fits piecewise form to sampling noise and should be recognized as such. Methods for estimating unknown knots, together with their well-documented sensitivity to specification and sample size, are treated in detail by Kohli et al. (2015), and random-knot extensions are developed by Feng et al. (2019).

Knot misspecification—placing the knot at the wrong time point—is methodologically treacherous: it produces apparently interpretable results while distorting the substantive conclusions, because the misspecification error is absorbed into the slope estimates rather than appearing as a model fit problem. Simulation work by Ram and Grimm (2007) suggests that reliable data-driven knot estimation requires a minimum of three measurement occasions per phase, and that with fewer than five total occasions, confidence intervals around estimated knot locations are typically too wide for precise inference.

An important advanced extension is the random knot model, in which the knot location itself is treated as a latent variable that varies across individuals. This is a substantially more demanding model, requiring a minimum of six to eight measurement occasions and *N* ≥ 200 for reliable estimation (Harring et al., 2006; Feng et al., 2019), but it produces a qualitatively richer picture of the heterogeneity of change processes.

### 4.3 Model identification and minimum data requirements

A two-piece linear model with a fixed knot requires a minimum of four measurement occasions for identification, with five or more strongly recommended. Simulation studies (e.g., Curran et al., 2010; see also Bollen and Curran, 2006) suggest minimum *N* of 100 for basic two-piece models, with larger samples required for growth mixture extensions. Researchers planning PLGM studies are encouraged to conduct a priori power analyses via Monte Carlo simulation, which can be implemented in Mplus using its MONTECARLO command or in R using the simsem package.

### 4.4 Extensions: growth mixture models and conditional PLGM

Growth Mixture Modeling (GMM; see Glossary) combined with piecewise specification allows the identification of latent subgroups following qualitatively different piecewise trajectories in response to the same intervention (Muthén and Muthén, 2000; Kohli et al., 2015). GMM solutions should be evaluated not only on statistical criteria (BIC, entropy) but on theoretical coherence: extracted classes must be interpretable in relation to the substantive theory of change guiding the intervention, and solutions that are statistically optimal but theoretically uninterpretable should be treated as artifacts rather than discoveries. Critically, the decision to adopt a mixture solution must rest on formal model comparison: a piecewise growth mixture model is justified only when it improves on a single-class piecewise model by appropriate criteria—information indices such as the BIC, the Lo–Mendell–Rubin likelihood-ratio test and the bootstrap likelihood-ratio test for the number of classes, and adequate entropy—and only when the resulting classes are theoretically interpretable. A mixture model that does not improve fit over its single-class counterpart adds complexity without warrant and should be rejected on that ground. Conditional PLGM incorporates time-varying and time-invariant covariates as predictors of both the phase-specific slopes and the knot location.

### 4.5 Relationship to other longitudinal models

The clearest way to state the distinction is this. A multilevel model treats the observed score at each occasion as the outcome and absorbs measurement error into the level-1 residual, whereas a latent-variable growth model represents the construct as latent and models measurement error explicitly at each occasion, so that the growth parameters are estimated free of attenuation. Both families can encode piecewise time structure; they differ in where measurement error is placed and, consequently, in what the model supports downstream—latent mediation of phase-specific change and, as the next subsection develops, formal testing of longitudinal measurement invariance. Multilevel models (MLM; Bryk and Raudenbush, 1987; Singer and Willett, 2003) can accommodate piecewise trajectories and offer practical advantages: they handle unbalanced data naturally, are widely implemented in accessible software, and require less specialist knowledge of structural equation modeling. For evaluation contexts in which measurement occasions are irregularly spaced or attrition is high, MLM with piecewise time coding may be the more practical choice. PLGM's distinctive advantages lie in its latent variable framework: it explicitly corrects for measurement error in each repeated measure, and its integration within the SEM family enables mediation of phase-specific change through latent intermediary variables. To make the selection concrete: a large-scale public health surveillance study with variable follow-up schedules and substantial dropout would favor MLM; a psychotherapy trial using a validated symptom instrument that specifies distinct mechanisms for early behavioral activation vs. later cognitive restructuring phases would favor PLGM. The choice should be governed by the theoretical demands of the evaluation rather than by analytic habit or software availability.

Table 1 summarizes the key choice criteria as a decision heuristic. Researchers should work through the questions in order; the first question that yields a definitive answer governs method selection.

**Table 1:** Decision heuristic for choosing between PLGM and MLM piecewise models.

Decision question	If YES	If NO	Notes
1. Are measurement occasions irregularly spaced or is attrition high?	Prefer MLM with piecewise coding	Proceed to Q2	MLM handles missingness via FIML by default
2. Is instrument reliability a documented concern (<0.80), or does the theory specify mechanisms (not just rates) of change?	Prefer PLGM (corrects for measurement error; supports mediation)	Proceed to Q3	PLGM's latent factor structure directly corrects slope attenuation
3. Is the evaluator's team proficient in SEM software (Mplus, lavaan, and OpenMx)?	Use PLGM	Use MLM or invest in SEM training before scaling up	Both can fit piecewise models; expertise is the practical constraint
4. Is identifying latent subgroups with qualitatively different trajectories a primary aim?	Use GMM-PLGM (*N* ≥ 150; treat as exploratory)	Standard PLGM with variance components is sufficient	Individual variance around slopes addresses heterogeneity without class extraction
5. Has the proposed model been compared against the next-simpler model (single-slope LGCM; or single-class PLGM for a mixture)?	Retain the more complex model only if it improves fit	Do not add complexity; report the simpler model	Applies at every step above: piecewise/mixture structure must earn its place by fit and theory

FIML, full information maximum likelihood; GMM, growth mixture model. Row 5 is added in this revision: model comparison is treated as an integral decision point rather than an optional final step.

### 4.6 PLGM and its alternatives: polynomial and exploratory models

Two further alternatives deserve direct comparison, because each represents a reasonable challenge to the recommendation of PLGM.

#### 4.6.1 Polynomial growth models

Where five or more occasions are available, a single-factor non-linear trajectory can be fitted by adding higher-order growth factors—a quadratic factor with loadings coded 0, 1, 4, 9, 16, or a cubic factor—rather than partitioning the trajectory into linear segments. Such models are often computationally simpler and can fit smoothly accelerating or decelerating processes well. They are, however, the appropriate choice for a specific and limited class of theories. A polynomial coefficient represents curvature; it does not represent a phase. Where the theory of change posits qualitatively distinct stages—Prochaska and DiClemente's (1983) stages of change, Kotter's (1996) sequential phases, the adoption-vs.-sustainment distinction of implementation science—the substantively meaningful quantities are the phase-specific rates and the locations of the transitions between them, and these are exactly what piecewise specification estimates and polynomial specification smooths away. The recommendation is therefore conditional on the theory: where the process is theorized as smoothly curvilinear, a polynomial LGCM is preferable; where it is theorized as phase-structured, PLGM is preferable; and the two should be compared on fit whenever the theory is ambiguous.

#### 4.6.2 Exploratory and network approaches

A second challenge comes from the observation that latent-variable growth models are heavily theory-laden: they presuppose a common latent trajectory generated by a small number of growth factors, and they may misrepresent phenomena better described as systems of mutually influencing components or as following heterogeneous, idiographic dynamics. Where these conditions obtain, less theory-committed approaches—dynamic network models, idiographic time-series methods—may be more faithful to the data-generating process. This is a genuine limitation of the present proposal, and it bounds its scope. PLGM is advanced here as the standard for evaluation research specifically: a domain defined by the prior specification of an intervention and a theory of how that intervention is expected to produce change over time. In that domain, the theory-ladenness of PLGM is a feature rather than a defect, because the evaluative task is precisely to test whether the theorized process occurred. In discovery-oriented longitudinal science, where the structure of change is itself the open question, exploratory methods have clear and legitimate priority. The claim of this paper is thus explicitly scoped and is not a claim about all longitudinal inquiry.

### 4.7 Longitudinal measurement invariance and second-order piecewise models

The measurement-theoretic concerns raised in Section 2—whether the same construct is being measured at each occasion—are not merely acknowledged within the latent-variable framework but are directly testable within it. This is one of PLGM's most consequential advantages over both the difference score and observed-variable multilevel models, and it answers the objection that growth models address only the structural portion of the model while leaving the measurement portion unexamined.

In a second-order, or curve-of-factors, piecewise growth model, the outcome at each occasion is itself a latent factor with multiple indicators, and the piecewise growth structure is estimated on those latent factors rather than on observed composites (Sayer and Cumsille, 2001). This specification permits formal testing of longitudinal measurement invariance: configural invariance (the same factor structure across occasions), metric invariance (equal factor loadings), and scalar invariance (equal indicator intercepts). Establishing at least partial scalar invariance is a precondition for interpreting phase-specific slopes as change in a construct of constant meaning, rather than drift in the relationship between the construct and its indicators. Where invariance fails, a second-order model localizes the failure to specific indicators and occasions instead of silently absorbing it, as a difference score necessarily does. This capability operationalizes the Borsboom (2005) requirement directly: it tests, rather than assumes, that variation in the latent construct is what produces variation in the observed scores across the full observation window. Neither difference scores nor observed-variable models can perform this test; for them, non-invariance simply becomes part of the reported “change.”

## 5 PLGM across applied domains: worked examples

Two interpretive notes apply to all four examples. First, the examples are illustrative: the parameter values are constructed to make the structural point transparent and are not reported as empirical findings. In a genuine analysis, each model would be accompanied by global fit indices (e.g., CFI, RMSEA, and SRMR) and by an explicit comparison against the simpler single-slope model, with the piecewise model retained only when it improves fit. Second, to keep the comparison fair, the difference scores discussed below are computed from the first and last occasions of the same multi-wave series used for the growth model (*D* = *T*_last_ – *T*_1_), not from a separate two-wave study. The point of each example is therefore not that a model with more occasions outperforms a study with fewer, but that—holding the data constant—a scalar displacement and a trajectory model extract different and non-substitutable information. Where an evaluation collects only two occasions, the trajectory information is simply unavailable, which is the rationale for the tiered framework of Section 6 rather than an argument against difference scores *per se*.

### 5.1 Clinical intervention: chronic disease management

Consider a 12-month lifestyle intervention for Type 2 diabetes management, with HbA1c measured at baseline, 3, 6, 9, and 12 months. Computing a difference score from the first and last of these same five occasions reports a mean reduction of 0.8% (*p* = 0.02) and would conclude the intervention is effective. A two-piece PLGM with a knot at 6 months reveals a Phase 1 slope of −0.22% per month (SE = 0.04, *p* < 0.001) and a Phase 2 slope of +0.09% per month (SE = 0.03, *p* = 0.003). The intervention produces meaningful early improvement, but participants are deteriorating in the second half. The difference score reported the right answer to the wrong question.

A GMM extension identifies three trajectory classes: sustained improvers (38%), relapsers (29%), and late responders (33%). These classes have different clinical profiles and likely different mechanisms of action—invisible to the difference score, which reports a single mean displacement. As above, this mixture solution would be adopted only after it improved on a single-class piecewise model by BIC, the bootstrap likelihood-ratio test, and entropy. GMM findings of this kind require replication in independent confirmatory samples before being used to guide treatment allocation or clinical policy decisions.

### 5.2 Psychotherapy evaluation: depression treatment

A 16-session CBT trial for major depressive disorder administers the PHQ-9 at sessions 1, 4, 8, 12, and 16. A difference score formed from sessions 1 and 16 of this series reports a mean reduction of 7.2 points and would conclude the treatment is effective. A two-piece PLGM with a theory-driven knot at session 8 estimates a Phase 1 slope of −1.4 points/session (SE = 0.18) and a Phase 2 slope of −0.3 points/session (SE = 0.14). The early phase accounts for over 80% of total symptom reduction, consistent with the early response literature (Howard et al., 1986).

In this illustrative case the Phase 2 slope is small and, given its standard error, would likely be statistically non-significant—a point that is itself instructive about model selection rather than an oversight. A near-zero second slope is evidence to be weighed against the two-piece model, not for it. The appropriate response is formal comparison: if a single decelerating trajectory (for example, a log-linear or asymptotic LGCM) reproduces these data as well as the two-piece model, parsimony favors the simpler form, and the piecewise specification earns its place only if it improves fit and the theory of change specifies a genuine phase boundary at session 8. Read this way, the example demonstrates not the automatic superiority of PLGM but the discipline of comparing it against simpler alternatives. The substantive lesson—that the bulk of symptom reduction is front-loaded—survives under either model. GMM-PLGM reveals that approximately one quarter of patients show a positive Phase 2 slope—worsening in the second half of treatment—a subgroup that would be identified as treatment responders by the pre–post criterion but who are in fact deteriorating at termination.

### 5.3 Curriculum evaluation: medical education

A 12-week interprofessional communication skills module is evaluated using a validated 40-item instrument (α = 0.82) at baseline, weeks 4, 8, 12, and 6-month follow-up. A difference score from baseline to week 12 within this five-occasion series reports a mean gain of 4.2 points (*p* < 0.01). A two-piece PLGM with a knot at week 8 estimates a Phase 1 slope of +0.84 points/week (SE = 0.12) and a Phase 2 slope of −0.21 points/week (SE = 0.09). A three-piece model incorporating the 6-month follow-up estimates a Phase 3 slope of −0.31 points/week, indicating near-complete decay to baseline. The pre–post gain of 4.2 points is real at the moment of measurement; it is not evidence of effective learning in any developmentally meaningful sense.

### 5.4 Organizational change: quality improvement implementation

A hospital-wide hand hygiene compliance initiative is evaluated over 18 months. A difference score formed from the first and last of the monitored occasions reports a 14-percentage-point improvement. A three-piece PLGM with knots at months 6 and 12 distinguishes implementation (+3.1 points/month), consolidation (+0.4 points/month), and institutionalization (−0.8 points/month) phases. The organization is actively deteriorating in the institutionalization phase—a finding that is actionable and invisible to the difference score.

## 6 Practical constraints and tiered implementation pathways

### 6.1 Acknowledging real-world constraints

The case for PLGM is theoretically compelling. Its practical adoption faces genuine obstacles: measurement frequency (most evaluation contexts collect fewer than four to five occasions), sample size (many studies operate with *N* < 50), statistical expertise, and funding cycles. These constraints are real but are often artifacts of institutional incentive structures rather than fixed features of the evaluation context.

The measurement-frequency constraint in particular deserves emphasis rather than minimization, because it is the single most common reason PLGM will be infeasible in a given study. Many—plausibly most—evaluation studies in the social and health sciences collect two or three occasions, and for these the full piecewise apparatus is simply unavailable: a two-piece fixed-knot model requires at least four occasions for identification, reliable estimation typically requires five or more, and data-driven or random knots require still more. I do not regard this as a refutation of the argument but as the reason the tiered framework below is structured as it is. The tiers are deliberately designed so that the lowest tier—two occasions—remains usable while making the interpretive limits of that tier explicit. The ontological argument sets the standard toward which designs should move as resources permit; it does not require that every study reach the highest tier, and it would be self-defeating to present a method most evaluators cannot apply as though it imposed a uniform requirement.

### 6.2 When difference scores are defensible: legitimate scope and appropriate use

Three contexts represent legitimate use cases for difference scores. First, formative evaluation contexts in which the question is scoped as a go/no-go decision may legitimately use a difference score when the pre–post correlation is low and interpretive claims are restricted to detection of displacement. Even in these contexts, residualized change scores are preferred over raw difference scores wherever baseline selection is non-random or regression-to-the-mean cannot be excluded. Second, routine continuous quality monitoring systems—weekly run charts, monthly compliance dashboards—function as control charts rather than evaluations, and adjacent point comparisons are appropriate for that purpose. The epistemological boundary is crossed when monitoring signals are reframed as evaluation evidence. Third, when the research question is explicitly concerned with group-level displacement at a specified time point and the pre–post correlation is demonstrably low, the difference score provides defensible evidence for that bounded question. In practice, most published studies that use difference scores report neither the correlation nor the reliability-of-difference-scores formula.

### 6.3 A tiered implementation framework

Table 2 presents a tiered framework matching analytic capacity to recommended approach. At every tier from Tier 2 upward, the recommended approach incorporates a formal model-comparison step: the proposed model is retained only if it improves on the next-simpler model by appropriate fit criteria (likelihood-ratio tests for nested models; information criteria and, for mixtures, the Lo–Mendell–Rubin and bootstrap likelihood-ratio tests for non-nested comparisons). Model selection is thus an integral decision point within each tier rather than a separate stage.

**Table 2:** Tiered implementation framework for trajectory-based evaluation.

Tier	Context	Recommended approach	Philosophical advance
1—Minimal	2 time points, small *N*, limited expertise	Residualized change scores; report pre–post correlation explicitly	Partial control for regression to the mean; honest reporting of reliability limits
2—Intermediate	3–4 time points, *N* ≥ 50	Single-slope LGCM with Bayesian estimation for small *N* (*N* = 50–99 without Bayesian: interpret with caution, report CIs)	Trajectory rather than displacement; individual differences in growth
3—Standard	4–5 time points, *N* ≥ 100	Two-piece PLGM with theory-driven knot	Phase-specific trajectory evidence; sustainability assessment
4—Comprehensive	5+ time points, *N* ≥ 150	Multi-piece PLGM or GMM-PLGM with conditional covariates	Latent trajectory classes; predictors of phase-specific change
5—Mixed-Methods	Any quantitative design + qualitative longitudinal data	PLGM + narrative interview or ethnographic observation at knot points	Quantitative trajectory grounded in phenomenological texture of change

Model selection is an integral decision point. At Tiers 2–5 the recommended model is adopted only if it improves on the next-simpler model by appropriate fit criteria: nested likelihood-ratio tests; information criteria such as the BIC; and, for mixture models, the Lo–Mendell–Rubin and bootstrap likelihood-ratio tests together with adequate entropy.

### 6.4 Bayesian approaches for resource-constrained settings

For settings where *N* < 100 precludes reliable frequentist PLGM estimation, Bayesian latent growth models offer a principled alternative. Bayesian PLGM makes its assumptions explicit in the form of priors and produces probability distributions over parameters rather than point estimates, which more transparently represents the uncertainty appropriate to small-sample evaluation. For frequentist PLGM, Mplus and the lavaan package in R both implement piecewise LGM; for Bayesian PLGM, the brms package in R allows piecewise growth models to be specified with informative priors. The combination of lavaan and brms constitutes a fully open-source, no-cost implementation pathway.

### 6.5 Qualitative longitudinal methods as complements

Where quantitative PLGM is infeasible, qualitative longitudinal methods—narrative interviews conducted at multiple time points, ethnographic observation across implementation phases, longitudinal case studies—can provide processual evidence of change philosophically coherent with the trajectory ontology. A concrete illustration: in a mixed-methods PLGM design evaluating an organizational quality improvement initiative, the quantitative trajectory model might identify the knot point—the specific month at which compliance rates transition from consolidation to decay. Researchers would then conduct semi-structured interviews with frontline staff at precisely that juncture, using the quantitatively identified inflection point as a theoretically grounded temporal anchor. Pettigrew's (1990) concept of receptive contexts for change is precisely the kind of construct that such qualitative longitudinal inquiry can operationalize, grounding abstract contextual variables in the lived experience of the implementation process.

## 7 A reporting framework for trajectory-based evaluation

We propose the following five-element framework for trajectory-based evaluation reporting.

### 7.1 Ontological declaration

Authors should explicitly state whether change is conceptualized as an event or a process, and justify that conceptualization in relation to the theoretical framework guiding the intervention. Guiding question: Is the change being studied best conceptualized as an event or a process? Justify your answer with explicit reference to your theoretical framework.

### 7.2 Temporal architecture justification

Authors should specify the number, spacing, and theoretical rationale for measurement occasions, and explain why that temporal architecture is adequate to characterize the trajectory of interest. Guiding question: How do your measurement points capture the full process of change, and why are they spaced appropriately? Where does your final measurement point fall in the hypothesized phase-structure?

### 7.3 Model–theory alignment

The chosen analytic model should be justified in relation to the theory of change, not merely statistical convention. Guiding question: How does your analytic model—including number of phases and knot placement—directly map onto your theory of change?

### 7.4 Displacement vs. trajectory distinction

Authors should explicitly distinguish between evidence of displacement and evidence of trajectory. Guiding question: Do your conclusions refer to a net displacement between two time points, or to a trajectory of change across time? Ensure that your interpretive language matches the evidence your design can actually support.

### 7.5 Model comparison and fit

Authors should report the global fit of the trajectory model and the explicit comparison between the adopted model and the simpler alternatives it is meant to improve upon—at minimum, the single-slope LGCM and, where a mixture model is used, the single-class solution. Where the measurement model is itself estimated (a second-order model), authors should report the longitudinal measurement-invariance tests that license the interpretation of change. Guiding question: What is the fit of your trajectory model; by what criteria does it improve on the simplest model consistent with your theory of change; and what evidence supports the invariance of your measurement model across occasions? Complexity beyond the simplest adequate model should be justified by fit, by theory, or by both.

## 8 Conclusions

The argument of this paper is unified by a single philosophical claim: evaluation research cannot produce interpretively valid evidence of change until it adopts analytic methods whose ontological commitments match the nature of the phenomena it studies. Change in clinical, therapeutic, educational, and organizational contexts is a process, not an event. Its theoretically important properties—phase structure, velocity, inflection, sustainability, heterogeneity—are invisible to any methodology that reduces it to a scalar displacement between two measurement points. This claim is bounded in two ways the preceding sections have made explicit: it applies to evaluation research, where an intervention and a theory of change are specified in advance, rather than to all longitudinal inquiry; and it presupposes, rather than supplies, a defensible and longitudinally invariant measurement model, without which neither a difference score nor a growth model can be interpreted.

Piecewise latent growth modeling is the operationalization of a better theory of change—one that the frameworks guiding intervention design already implicitly require but that evaluative practice has consistently failed to honor. The tiered framework proposed here provides implementation pathways that do not require all evaluation contexts to achieve the same analytic ceiling. What the framework does require, at every tier, is epistemic honesty: the recognition that a pre–post comparison establishes displacement and nothing more, and the discipline of retaining no more model complexity than fit and theory jointly justify.

The difference score has served the field long enough. The field of evaluation must evolve—from relying on oversimplified difference scores to embracing trajectory-sensitive models like PLGM that can truly capture the complexity of change processes. Evaluation research that asks only “Did it work?” will continue to produce answers that are technically correct and substantively misleading. The standard must be “How did it unfold?”—because only that question produces evidence worthy of the name.

## Data Availability

The original contributions presented in the study are included in the article/supplementary material, further inquiries can be directed to the corresponding author/s.

## Appendix

### Glossary of key terms

**Difference score (*D* = Post – Pre)**. The arithmetic subtraction of a pre-intervention score from a post-intervention score. Subject to systematic reliability degradation when the pre–post correlation is high relative to component reliabilities. Should not be interpreted as a trajectory-level index of change.

**Growth factor (intercept and slope)**. Latent variables in a growth model. The intercept factor represents the estimated level of the outcome at a reference time point; the slope factor represents the rate of change per unit of time. Both have means and variances representing average trajectory parameters and individual differences.

**Growth mixture model (GMM)**. An extension of latent growth curve modeling that identifies latent subgroups of individuals who follow qualitatively different trajectory patterns (see Muthén and Muthén, 2000). GMM solutions are exploratory, should be interpreted as hypothesis-generating rather than confirmatory, should be adopted only when they improve on a single-class solution by appropriate model-comparison criteria, and require replication in confirmatory samples.

**Knot point (κ)**. The defined transition point in a piecewise growth model at which one trajectory segment ends and another begins. Correct knot specification is critical: misspecification distorts phase-specific slope estimates in ways that may not be detectable from overall model fit indices alone.

**Latent growth curve model (LGCM)**. A statistical model within the structural equation modeling (SEM) family that represents individual change by estimating latent growth factors from repeated measurements. Unlike MLM, LGCM explicitly models measurement error in each repeated observation, producing unattenuated growth parameter estimates.

**Longitudinal measurement invariance**. The property that a measurement model relates a latent construct to its indicators in the same way across measurement occasions. Configural, metric, and at least partial scalar invariance are preconditions for interpreting estimated change as change in a construct of constant meaning. Testable within second-order latent growth models, but not within difference scores or observed-variable multilevel models.

**Multilevel model (MLM)**. Also called hierarchical linear model (HLM) or mixed-effects model. Estimates fixed and random effects for longitudinal data, treating observed scores as the dependent variable and absorbing measurement error into the residual. Handles unbalanced data and irregular measurement spacing more flexibly than SEM-based approaches.

**Phase-specific slope**. The estimated rate of change within a single defined phase of the observation period in a piecewise latent growth model. Phase-specific slopes are the primary substantive output of PLGM analysis.

**Piecewise latent growth model (PLGM)**. An extension of LGCM that models change as a sequence of two or more distinct trajectory segments, each with its own slope, joined at one or more knot points. Can be extended to GMM-PLGM for subgroup trajectory analysis or to random knot models for heterogeneous transition timing.

**Processual ontology**. A philosophical position that treats change as a continuous, temporally extended trajectory rather than a discrete transition between states. Evaluation methods calibrated to characterize trajectories rather than displacements are required under this ontology.

**Residualized change score**. A post-intervention score adjusted for the pre-intervention score such that the residual represents change beyond what baseline levels predicted. Constitutes the minimum adequate improvement over D = Post – Pre in two-wave designs but cannot characterize a trajectory.

**Second-order (curve-of-factors) growth model**. A growth model in which the outcome at each occasion is itself a latent factor with multiple indicators and the growth structure is estimated on those latent factors. Permits longitudinal measurement-invariance testing and is the appropriate vehicle for PLGM when the measurement model must be examined rather than assumed.
